# Early changes in health coverage and access to dental care associated with Medicaid expansion under the COVID-19 pandemic

**DOI:** 10.1093/haschl/qxad032

**Published:** 2023-08-01

**Authors:** Hawazin W Elani, Jose F Figueroa, Ichiro Kawachi, Meredith Rosenthal

**Affiliations:** Department of Oral Health Policy and Epidemiology, Harvard School of Dental Medicine, Boston, MA 02115, United States; Department of Health Policy and Management, Harvard T.H. Chan School of Public Health, Boston, MA 02115, United States; Department of Health Policy and Management, Harvard T.H. Chan School of Public Health, Boston, MA 02115, United States; Department of Social and Behavioral Sciences, Harvard T.H. Chan School of Public Health, Boston, MA 02115, United States; Department of Health Policy and Management, Harvard T.H. Chan School of Public Health, Boston, MA 02115, United States

**Keywords:** COVID-19, health coverage, Medicaid expansion, Patient Protection and Affordable Care Act, oral health, health care disparities, National Health Interview Survey, dental insurance, dental health services

## Abstract

The extent to which the COVID-19 pandemic has affected early changes in health coverage and access to dental care services in states that expanded Medicaid versus those that did not is currently not well known. Using data from the National Health Interview Survey, we found that, during the first year of the COVID-19 pandemic, states that had previously expanded their Medicaid programs under the Affordable Care Act had lower uninsurance rates for White low-income adults (−8.8 percentage points; 95% CI: −16.6, −1.0) and lower dental uninsurance rates for all low-income adults (−5.4 percentage points; 95% CI: −10.4, −0.5). Our findings also suggest that the combination of Medicaid expansion with coverage of adult dental benefits in Medicaid was associated with improved dental coverage and access to dental care during the pandemic. With the expiration of the public health emergency declaration, states are considering strategies to prevent disruptions in Medicaid coverage. Our study adds to the evidence of the importance of Medicaid expansion in stabilizing health coverage during a public health crisis.

## Teaser

In this study, we used national data and leveraged state-level variations in Medicaid expansion and coverage of adult dental benefits to examine whether the Medicaid expansion stabilized health coverage and access to dental care during the first year of the COVID-19 pandemic, and whether the combination of Medicaid expansion with coverage of adult dental benefits in Medicaid improved dental outcomes. Our findings suggest that Medicaid expansion mitigated the effect of the COVID-19 pandemic on health insurance for White low-income adults only, but reassuringly, with lower dental uninsurance rates for all low-income adults. We also found that the combination of Medicaid expansion with coverage of adult dental benefits in Medicaid was associated with improved dental coverage and access to dental care during the pandemic.

## Introduction

Since the enactment of the Patient Protection and Affordable Care Act (ACA), 40 states and the District of Columbia have expanded Medicaid to include previously uncovered low-income adults (hereafter “the Medicaid expansion”) and more than 35 million Americans have gained health coverage.^1,2^ Research over the past decade has documented that the ACA improved access to health care and health outcomes, including oral health.^3–5^ On the other hand, although evidence suggests that the ACA produced gains in dental coverage,^6^ lowered use of nonurgent emergency department dental use,^7^ and improved several indicators of oral health,^5,8^ coverage of adult dental benefits remains an optional benefit for state Medicaid programs.

The coronavirus disease 2019 (COVID-19) pandemic was a test of the effectiveness of the ACA in protecting vulnerable populations during a public health emergency and economic recession.^9^ The pandemic-related recession led to massive job losses, contributing to sharp declines in employer-sponsored health insurance.^10^ To date, there has been substantial evidence to suggest that COVID-19 disrupted health coverage and access to health care^11,12^; however, it remains unclear at a national level whether the Medicaid expansion stabilized health coverage during the pandemic.^13^ Even less is known about how the pandemic affected access to dental care services, especially among racial and ethnic minority and low-income populations that historically have lower rates of dental visits and poorer oral health. Therefore, using national data and leveraging state-level variations in Medicaid expansion and coverage of adult dental benefits, we estimated early changes in insurance coverage and access to oral health services during the first year of the COVID-19 pandemic.

## Data and methods

### Data and study sample

We analyzed data from 2016 to 2020 from the National Health Interview Survey (NHIS), which is conducted annually by the National Center for Health Statistics.^14^ We obtained access to restricted state identifiers through Federal Statistical Research Data Centers. To describe trends in outcomes over time, we included NHIS data from 2010 to 2020. We restricted the sample to low-income adults ages 19 to 64 years. The NHIS reports family income in predefined categories. In the primary analysis we used the lowest income category (below 125% of the Federal Poverty Level [FPL]) to define our low-income sample. In sensitivity analyses, we used imputed family income files provided by the National Center for Health Statistics to define a low-income sample up to 138% of the FPL.

### Primary outcomes

Health insurance coverage outcomes included Medicaid and uninsured status. Dental insurance coverage outcomes included Medicaid, uninsured, and private dental coverage. Measures of access to dental care were having a dental visit in the past year and inability to afford dental care in the past year. All variables were self-reported and binary indicators.

### Statistical analysis

We used a difference-in-difference linear regression to compare changes in outcomes in expansion states and non-expansion states before and after the onset of the COVID-19 pandemic. Our models controlled for age, sex, education, race/ethnicity, marital status, citizenship, number of children, state-year unemployment rate,^15^ number of dentists per capita in each state,^16^ state-level COVID-19 rates during 2020,^17^ year, and state. To account for correlation among the error terms within a state, we used robust standard errors clustered by state^18^ (regression details are shown in Appendix S7). We examined the full sample and then used the same regression model to separately examine states that do and do not provide Medicaid adult dental benefits. We defined a state as providing adult dental benefits in Medicaid if it offered more than emergency dental coverage to Medicaid beneficiaries (Table S1).^5,6^ We examined racial and ethnic disparities in outcomes by repeating the main analysis but stratifying according to respondents’ race and ethnicity.

Finally, to examine how state Medicaid policy affected dental outcomes during the first year of the COVID-19 pandemic, we compared the prevalence of dental insurance coverage and access to dental care outcomes across 4 state groups based on whether they expanded Medicaid and whether their Medicaid programs provide dental benefits. We used multivariate logistic regression for each outcome and generated predicted probabilities using marginal standardization. We used cross-sectional data from the year 2020 and adjusted models for age, sex, education, race/ethnicity, family income, marital status, state-level COVID-19 rates during 2020,^17^ and state.

### Sensitivity analyses

We excluded states that expanded the ACA in 2019 and 2020, excluded states that changed their dental benefits between 2016 and 2020, and used imputed family income files to limit the sample to adults with a family income up to 138% of the FPL. We also examined differences in outcomes in expansion and non-expansion states before 2020 by examining trends in outcomes according to expansion status and by conducting a falsification test using data from 2016 to 2019 and the year 2019 as the placebo COVID year. We used NHIS survey weights to account for the complex survey design and Stata software version 15.1 for all analyses.^19^

## Results

### Sample characteristics

Our unweighted sample included 26 337 low-income adults. The demographic characteristics of the sample at baseline (from 2016 to 2019) are presented in Table S2. Low-income adults in non-expansion states were more likely to be non-Hispanic Black (27.2% vs 18.0%) and to have less than a high school education (35.7% vs 31.4%) than those in expansion states.

### Changes in coverage and access to dental care during the COVID-19 pandemic in association with state Medicaid expansion

In 2020, there were no significant changes (3.3 percentage points; 95% CI: −5.0, 11.6) in Medicaid coverage and in the uninsurance rates in expansion states versus non-expansion states compared with before the COVID-19 pandemic (Table 1). However, in subgroup analysis, we found that the proportion of uninsured White adults significantly declined in expansion states (−8.8 percentage points; 95% CI: −16.6, −1.0) relative to non-expansion states (Table S3).

**Table 1.** Changes in outcomes among low-income adults after the COVID-19 pandemic in association with state Medicaid expansion in the full sample and according to states' dental benefits status.

	Baseline before COVID-19 (mean % in expansion states)^a^		Differences-in-differences, net change after 2020^b^	
Outcome	%	95% CI	%	95% CI
Medicaid coverage				
Full sample	51.2	(49.5, 53.0)	3.3	(−5.0, 11.6)
States without dental benefits	40.8	(35.4, 46.4)	6.2	(−6.9, 19.2)
States with dental benefits	52.7	(50.9, 54.5)	1.0	(−8.7, 10.8)
Uninsured				
Full sample	17.9	(16.8, 19.1)	−7.7	(−16.6, 1.1)
States without dental benefits	26.5	(23.2, 30.2)	−7.0	(−24.3, 10.3)
States with dental benefits	16.7	(15.5, 17.9)	−6.9	(−18.7, 4.9)
Medicaid dental coverage				
Full sample	46.2	(43.9, 48.5)	2.1	(−1.6, 5.7)
States without dental benefits	—	—	—	—
States with dental benefits	52.7	(50.9, 54.5)	1.0	(−8.7, 10.8)
Private dental insurance				
Full sample	17.9	(16.8, 19.1)	3.1	(−1.2, 7.3)
States without dental benefits	18.7	(15.2, 22.8)	0.8	(−8.7, 10.3)
States with dental benefits	17.8	(16.6, 19.0)	2.1	(−4.8, 9.1)
No dental coverage				
Full sample	39.6	(37.6, 41.7)	−5.4	(−10.4, −0.5)**
States without dental benefits	81.3	(77.2, 84.8)	−0.8	(−10.3, 8.7)
States with dental benefits	33.7	(32.4, 35.2)	−4.3	(−15.1, 6.6)
Seen a dentist in the past year^c^				
Full sample	51.5	(48.4, 54.6)	−12.3	(−23.6, −1.0)**
States without dental benefits	39.2	(31.8, 47.1)	−15.8	(−42.3, 10.7)
States with dental benefits	53.4	(50.1, 56.6)	−1.9	(−21.0, 17.2)
Couldn’t afford dental care in the past year^c^				
Full sample	21.2	(18.8, 23.8)	3.6	(−11.0, 18.1)
States without dental benefits	32.2	(25.0, 40.4)	−1.4	(−25.4, 22.6)
States with dental benefits	19.6	(17.1, 22.3)	6.7	(−11.9, 25.2)

Source: Authors’ analysis of data from the National Health Interview Survey (NHIS) from 2016 to 2020. The study sample limited to adults ages 19–64 years with an income below 125% of the Federal Poverty Level (n = 26 337). **P < .05.

a Baseline refers to the sample prior to COVID-19 (NHIS survey data from the period 2016–2019).

b Model adjusted for age, sex, race/ethnicity, education, marital status, citizenship, number of children, state-year unemployment rate, number of dentists per capita in each state, state-level COVID-19 rates during 2020, year, and state. All analyses used robust standard errors clustered by state.

c Sample restricted to respondents with interviews in the last quarter of 2020 because these questions had a 12-month look-back period, which mostly occurred before the pandemic (n = 3400 and n = 3445, respectively). States that did and did not expand Medicaid during our study period and that do and do not provide adult Medicaid dental benefits are listed in Table S1.

Furthermore, we found a 5.4-percentage-point (95% CI: −10.4, −0.5) decline in the dental uninsurance rate in expansion states versus non-expansion states in 2020 for all low-income adults, but we did not detect any significant changes in Medicaid or private dental coverage. We also found that the pandemic was associated with significant reductions in the proportion of low-income adults reporting seeing a dentist in the past year in expansion states (−12.3 percentage points; 95% CI: −23.6, −1.0) compared with non-expansion states. There were no significant changes in the affordability of dental care between expansion and non-expansion states.

Unadjusted trends in outcomes according to expansion status are presented in Figure S1. Results from the placebo analysis provided support to our difference-in-difference design (Table S4). Our findings were also generally robust to several sensitivity checks using alternative sample definitions (Tables S5 and S6).

### Rates of dental coverage and access to dental care in 2020

**Figure 1.**
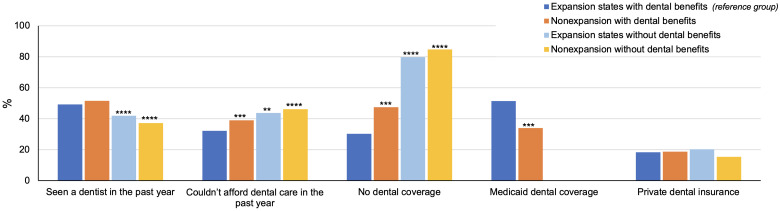
Dental coverage and access to dental care in the year 2020 among low-income adults in Medicaid expansion and non-expansion states by state dental benefit status. Source: Authors’ analysis of data from the National Health Interview Survey, year 2020. The study sample was limited to adults ages 19–64 years with an income below 125% of the Federal Poverty Level (n = 2782). Results are from survey-weighted logistic regression models adjusted for age, sex, education, race/ethnicity, family income, marital status, state-level COVID-19 rates during 2020, and state. States that did and did not expand Medicaid during our study period and that do and do not provide adult Medicaid dental benefits are listed in Table S1. **P < .05; ***P < .01; ****P < .001.

Comparisons of dental outcomes in 2020 across the 4 state groups based on Medicaid expansion and coverage of adult dental benefits are presented in Figure 1. In terms of dental coverage, rates of Medicaid coverage were significantly higher in expansion states that provide adult dental benefits compared with non-expansion states with adult dental benefits (51.4% vs 34.0%; *P* < .01). We found that rates of dental uninsurance were significantly lower in expansion states that provide adult dental benefits (30.3%) compared with the remaining 3 state groups (non-expansion states with dental benefits [47.4%; *P* < .01], expansion states without dental benefits [79.7%; *P* < .001], and non-expansion states without dental benefits [84.6%; *P* < .001]). We did not find any significant differences in private dental coverage across the 4 state groups.

Expansion and non-expansion states without dental benefits had significantly lower rates of dental visits compared with states providing dental benefits. Rates of inability to afford dental care in the previous year were significantly lower in expansion states that provide adult dental benefits (32.2%) compared with non-expansion states with dental benefits (38.9%; *P* < .01), expansion states without dental benefits (43.7%; *P* < .05), and non-expansion states without dental benefits (46.1%; *P* < .001).

## Discussion

Our study provides insights into the early impact of the COVID-19 pandemic on changes in health coverage and use of dental services. We found that Medicaid expansion was associated with lower uninsurance rates for White low-income adults only, but reassuringly, with lower dental uninsurance rates for all low-income adults. Our findings also suggest that the combination of Medicaid expansion with coverage of adult dental benefits in Medicaid was associated with improved dental coverage and access to dental care during the pandemic.

Although we observed increases in Medicaid enrollment and reductions in the uninsurance rates overall, we did not find significant differences between expansion and non-expansion states during the first year of the pandemic. These findings are in line with early studies during the pandemic indicating growth in Medicaid enrollment in both expansion and non-expansion states.^2,20,21^ This is likely related to the maintenance of eligibility coverage provisions under the Families First Coronavirus Recovery Act (FFCRA) that prevented Medicaid disenrollment and churning during the public health emergency in all states, regardless of their expansion status.^22–24^ However, we observed statistically significant reductions in uninsurance rates in expansion states relative to non-expansion states for White adults. Racial and ethnic minority populations experienced disproportionate rates of COVID-19 infection and mortality^25^ and thus may have deferred coverage decisions. It is also possible that newly unemployed, uninsured minority adults, who are Medicaid eligible, were unaware of their coverage options, which likely resulted in low uptake of coverage.^26^

With regard to dental care, we found that the expansion was associated with reductions in dental uninsurance rates during the first year of the pandemic. This was driven by an increase in dental uninsurance in non-expansion states. Consistent with prior evidence from other health care use studies,^27^ we also found that the expansion was associated with reductions in access to dental care. Along with the closure of dental clinics due to the pandemic, fear of exposure to COVID-19 caused many adults to forgo routine dental care. These results have important implications for the dental care system as it recovers from the pandemic. Even though the extent to which these disruptions may have impacted oral health is not yet clear, the increased demand from patients awaiting care will add strain on the dental care system, which is already experiencing challenges to serve the low-income population.^28^

In our cross-sectional state comparison, we observed that respondents in expansion states that cover dental benefits reported the highest rates of Medicaid dental coverage and the lowest rates of dental uninsurance compared with non-expansion states and expansion states without dental benefits. Similarly, we found higher rates of dental visits and lower rates of cost-associated barriers to dental care in states that provide adult dental coverage in Medicaid. While the pandemic created substantial challenges to accessing dental services, it appears that coverage of adult dental benefits in Medicaid in combination with the expansion has reduced barriers to accessing dental care for low-income adults during the pandemic.

### Limitations

First, during the COVID-19 pandemic, NHIS shifted from in-person to telephone interviews, raising risks for nonresponse bias.^29^ However, we used survey weights as recommended by NHIS to mitigate this risk. Second, our analysis focused on the first year after the pandemic, which only captures the early effect of COVID-19. Third, for access to dental care measures, we restricted our sample to respondents with interviews in the last quarter of 2020 because these questions had a 12-month look-back period, which mostly occurred before the pandemic. This may have limited our statistical power to detect pandemic-associated changes, as indicated by our wide 95% CIs. Finally, we did not examine the impact of the provisions for maintaining eligibility coverage under the FFCRA during the pandemic. However, this provision affected both expansion and non-expansion states and thus may have skewed our results toward the null.

## Conclusion

With the expiration of the public health emergency declaration, states are considering strategies to prevent disruptions in Medicaid coverage. Our study adds to the evidence of the importance of Medicaid expansion in stabilizing health coverage during a national public health emergency. Further, we highlight the importance of dental coverage in protecting low-income populations during an economic crisis to inform future policy responses.

## Supplementary Material

qxad032_Supplementary_Data
