# Supplementary material for: Early changes in health coverage and access to dental care associated with Medicaid expansion under the COVID-19 pandemic
Source: Health Aff Sch. Author manuscript; Available in PMC 2024 Mar 18. (PMC10948102; doi:10.1093/haschl/qxad032)
Supplement: supplementary data [file NIHMS1928429-supplement-supplementary_data.docx]

**Supplementary Online Content**

**eTable 1.** Classification of states included in the study according to Medicaid expansion and dental benefits status.

**eTable 2.** Demographic characteristics of low-income adults in Medicaid expansion and control states at baseline (Years NHIS: 2016-2019).

**eFigure 1.** Unadjusted trends in outcomes according to Medicaid expansion status.

**eTable 3.** Changes in outcomes among low-income adults after the COVID-19 pandemic in association with state Medicaid expansion by race and ethnicity.

**eTable 4.** Robustness Checks- Changes in outcomes among low-income adults using NHIS data from 2016 to 2019 and year 2019 as the placebo COVID year.

**eTable 5.** Sensitivity analyses according to state Medicaid expansion in the full sample and according to state’s dental benefits status.

**eTable 6.** Sensitivity analyses according to race and ethnicity.

**Appendix 7.** Methods, Regression Equation.

**eTable 1.** Classification of states included in the study according to Medicaid expansion and dental benefits status.

|  | **States** |
| --- | --- |
| **Medicaid expansion states** | AK,AZ, AR, CA, CO, CT, DE, DC, HI, ID, IL, IN, IA, KY, LA, ME, MD, MA, MI, MN, MT, NE, NV, NH, NJ, NM, NY, ND, OH, OR, PA, RI, UT, VT, VA, WA, WV. |
| **Medicaid non-expansion states** | AL, FL, GA, KS, MO, MS, NC, OK,SC, SD, TN, TX, WI, WY. |
| **Medicaid expansion states with dental benefits** | AK, AR, CA, CO, CT, DC, HI, IA, ID, IL, IN, KY, LA, MA, MI, MN, MT,ND, NE, NJ,NM, NY, OH, OR, PA, RI, VT, WA,WV. |
| **Medicaid expansion states without dental benefits** | AZ, DE,MD,ME,NH,NV, UT,VA. |
| **Medicaid non-expansion states with dental benefits** | KS, MO, NC, SC, SD, WI, WY. |
| **Medicaid non-expansion states without dental benefits** | AL, FL, GA, MS, OK,TN, TX. |

***Source:***

*1. Kaiser Family Foundation; Status of State Medicaid Expansion Decisions. Available from: https://www.kff.org/medicaid/issue-brief/status-of-state-medicaid-expansion-decisions-interactive-map/. Accessed March 13, 2019.*

*2. Centers for Medicare and Medicaid Services. Medicaid State Plan Amendments. Available from: https://www.medicaid.gov/state-resource-center/medicaid-state-plan-amendments/index.html. Accessed March 13, 2019.*

*3. Center for Health Care Strategies. Medicaid adult dental benefits: An overview. 2019. Available from: https://www.chcs.org/resource/medicaid-adult-dental-benefits-overview/. Accessed April 10, 2020*

**eTable 2.** Demographic characteristics of low-income adults in Medicaid expansion and control states at baseline (Years NHIS: 2016-2019).

|  | **Expansion states**  **( n=14,838)** | **Non-Expansion states**  **(n= 8,717)** |
| --- | --- | --- |
|  | **n, (weighted %)** | **n, (weighted %)** |
| **Variable** |  |  |
| **Gender** |  |  |
| **Male** | 4,865 (44.8) | 2,165 (43.5) |
| **Female** | 6,186 (55.2 ) | 3,113 (56.5) |
|  |  |  |
| **Age** |  |  |
| **Average age (±SD)** | 38.4 (±13.9) | 38.6 (±13.6) |
| **19-34** | 6,378 (45.1) | 3,623 (45.0) |
| **35-44** | 2,886 (19.6) | 1,806 (20.2) |
| **45-54** | 2,668 (17.5) | 1,539 (16.5) |
| **55-64** | 2,906 (17.8) | 1,749 (18.3) |
|  |  |  |
| **Race and ethnicity** |  |  |
| **Non-Hispanic White** | 7,323 (45.9) | 3,469 (38.5) |
| **Non-Hispanic Black** | 2,335 (18.0) | 2,279 (27.2) |
| **Hispanic** | 3,658 (27.7) | 2,463 (30.1) |
| **Other ^a^** | 1,087 (8.4) | 314 (4.2) |
|  |  |  |
| **Education** |  |  |
| **Less than High school** | 4,475 (31.4) | 3,069 (35.7) |
| **High school graduate** | 3,780 (26.0) | 2,294 (27.0) |
| **Greater than high school** | 6,391 (42.5) | 3,251 (37.3) |
|  |  |  |
| **Marital status** |  |  |
| **Single** | 10,233 (69.0) | 5,983 (67.9) |
| **Married** | 4,485 (31.0) | 2,689 (32.1) |
|  |  |  |
| **Citizenship** |  |  |
| **Foreign** | 2,319 (18.1) | 1,511(18.7) |
| **US Citizen** | 12,368 (81.9) | 7,135 (81.3) |
|  |  |  |
| **Nativity** |  |  |
| **Foreign** | 3,569 (27.4) | 2,056 (25.5) |
| **US-born** | 11,145 (72.6) | 6,615 (74.5) |

**Source.** Authors’ analysis of data from the National Health Interview Survey from 2016 to 2019. **Note:** Sample limited to adults ages 19-64 with family income below 125 percent of the federal poverty level. ^a^Other include, Indian (American), Asian, and other race. States that did and did not expand Medicaid during our study period are listed in eTable 1 in the Supplement.

**eFigure 1.** Unadjusted trends in outcomes according to Medicaid expansion status.

**
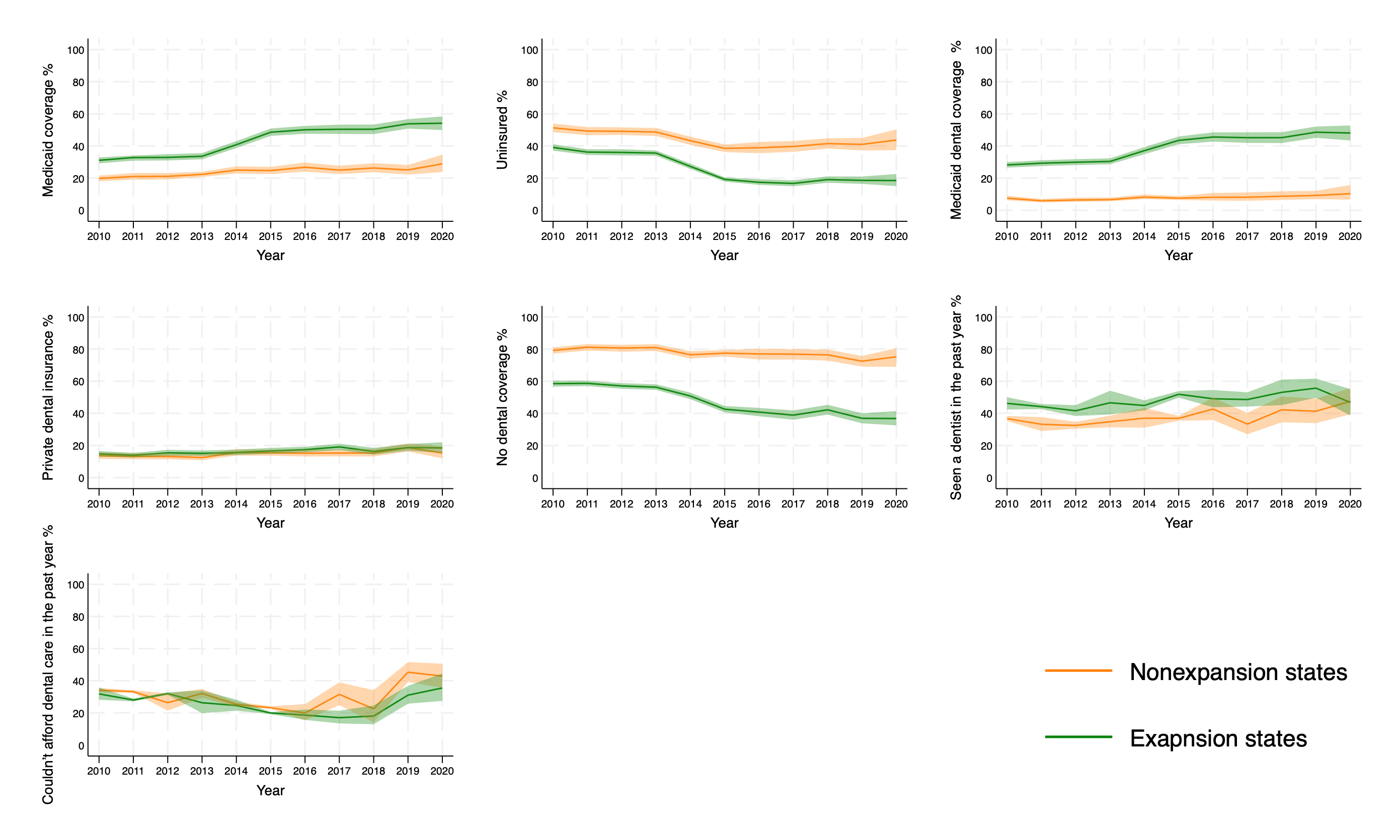
**

**Source.** Authors’ analysis of data from the National Health Interview Survey from 2010 to 2020. Notes: Study sample limited to adults ages 19–64 with income below 125 percent of the federal poverty level (n= 93,640). States that did and did not expand Medicaid during our study period are listed in eTable 1 in the Supplement.

**eTable 3.** Changes in outcomes among low-income adults after the COVID-19 pandemic in association with state Medicaid expansion by race and ethnicity.

|  | **Baseline before COVID-19**  **(mean % in expansion states) ^a^** | | **Differences-in-Differences** | |
| --- | --- | --- | --- | --- |
|  |  |  | **Net change after 2020 ^b^** | |
| **Outcome** |  | |  | |
|  | **%** | **95% CI** | **%** | **95% CI** |
| **Medicaid coverage** |  |  |  |  |
| White | 49.8 | (47.5, 52.1) | 3.6 | (-5.3,12.6) |
| Black | 60.7 | (57.8, 63.5) | -6.3 | (-26.6, 13.9) |
| Hispanic | 47.3 | (44.5, 50.1) | 3.1 | (-6.9, 13.1) |
|  |  |  |  |  |
| **Uninsured** | |  |  |  |
| White | 12.1 | (10.9, 13.5) | -8.8 | (-16.6, -1.0)** |
| Black | 13.5 | (11.4,15.9) | 0.5 | (-17.5, 18.5) |
| Hispanic | 32.0 | (29.4, 34.8) | -5.6 | (-15.7, 4.6) |
|  |  |  |  |  |
| **Medicaid dental coverage** | |  |  |  |
| White | 45.1 | (42.4, 47.8) | 0.7 | (-5.1, 6.5) |
| Black | 52.6 | (47.9, 57.3) | 0.7 | (-7.5, 9.0) |
| Hispanic | 42.8 | (39.3, 46.3) | 0.5 | (-8.8, 9.8) |
|  |  |  |  |  |
| **Private dental insurance** | |  |  |  |
| White | 22.0 | (20.3, 23.8) | 6.2 | (-0.8, 13.2) |
| Black | 15.2 | (13.3, 17.2) | -3.0 | (-14.4, 8.5) |
| Hispanic | 12.8 | (11.3, 14.4) | 3.9 | (-4.9, 12.8) |
|  | |  |  |  |
| **No dental coverage** | |  |  |  |
| White | 36.2 | (33.9, 38.5) | -7.3 | (-15.2, 0.6) |
| Black | 36.6 | (32.6, 40.8) | 1.8 | (-8.4, 12.1) |
| Hispanic | 48.3 | (44.9, 51.8) | -6.0 | (-17.3, 5.3) |
|  |  |  |  |  |
| **Seen a dentist in the past year ^c^** |  |  |  |  |
| White | 52.8 | (48.9, 56.6) | -12.9 | (-29.2, 3.3) |
| Black | 53.8 | (46.3,61.1) | -37.9 | (-80.0, 4.1) |
| Hispanic | 49.0 | (41.4, 56.6) | 17.9 | (-8.2, 44.1) |
|  |  |  |  |  |
| **Couldn’t afford dental care in the past year ^c^** |  |  |  |  |
| White | 22.6 | (19.3, 26.3) | 12.8 | (-5.4, 31.1) |
| Black | 17.5 | (12.7, 23.6) | 10.6 | (-19.0, 40.3) |
| Hispanic | 23.5 | (18.5, 29.3) | -17.8 | (-39.4. 3.8) |

**SOURCE**. Authors’ analysis of data from the National Health Interview Survey from 2016 to 2020. **Notes**: Study sample limited to adults ages 19–64 with income below 125 percent of the federal poverty level. ^a^ Baseline refers to the sample prior to COVID-19 (NHIS survey data from the period 2016-2019). ^b^ Model adjusted for age, sex, education, marital status, citizenship, number of children, state-year unemployment rate, number of dentists per capita in each state, state-level COVID-19 rates during 2020, year, and state. All analyses used robust standard errors clustered by state. ^c^ Sample restricted to respondents with interviews in the last quarter of 2020 because these questions had a 12-month look-back period which mostly occurred before the pandemic. We excluded “Other” race/ethnicity group that includes, Indian (American), Asian, and other race due to limited statistical power. States that did and did not expand Medicaid during our study period are listed in eTable 1 in the Supplement. CI=confidence interval. ***p*< 0.05.

**eTable 4.** Robustness Checks-Changes in outcomes among low-income adults using NHIS data from 2016 to 2019 and year 2019 as the placebo COVID year.

|  | **Differences-in-Differences** | | | | | | | | | | | | | |
| --- | --- | --- | --- | --- | --- | --- | --- | --- | --- | --- | --- | --- | --- | --- |
|  | **Net change after 2019 (%, 95% CI)** | | | | | | | | | | | | | |
| **Outcome** | **Medicaid Coverage** | | **Uninsured** | | **Medicaid dental coverage** | | **Private dental insurance** | | **No dental coverage** | | **Seen a dentist in the past year** | | **Couldn’t afford dental care in the past year** | |
| **Full sample** | 2.0 | (-3.1, 7.2) | 0.1 | (-4.6, 4.8) | 0.5 | (-3.4, 4.3) | -0.5 | (-4.6, 3.6) | 1.5 | (-3.7, 6.8) | 2.1 | (-7.9, 12.2) | -7.0 | (-14.0, 0.1) |
| **States without dental benefits** | 5.6 | (-7.3, 18.5) | -0.7 | (-7.9, 6.5) | -- | -- | 0.2 | (-9.2, 9.7) | -0.2 | (-9.7, 9.2) | 1.6 | (-17.5, 20.7) | 12.2 | (-6.9, 31.3) |
| **States with dental benefits** | -0.8 | (-8.4, 6.7) | 1.3 | (-2.9, 5.5) | -0.8 | (-8.4, 6.7) | 4.6 | (1.2, 8.1)** | -3.2 | (-9.0, 2.5) | 7.8 | (-5.7, 21.3) | -11.0 | (-22.0, 0.0) |
| **White** | 5.5 | (-0.4, 11.5) | -0.5 | (-6.0, 5.0) | 3.0 | (-2.0, 7.9) | -6.0 | (-14.1, 2.2) | 2.3 | (-6.8, 11.3) | -13.3 | (-29.1, 2.6) | -12.3 | (-25.9, 1.3) |
| **Black** | 2.4 | (-7.4, 12.2) | 1.9 | (-7.3, 11.1) | 4.4 | (-2.8, 11.7) | 1.4 | (-4.6, 7.4) | -3.0 | (-10.4, 4.3) | 3.8 | (-18.1,25.7) | -15.5 | (-38.7, 7.7) |
| **Hispanic** | 0.2 | (-7.2, 7.6) | 0.6 | (-7.6, 8.8) | -3.2 | (-7.6, 1.2) | 3.7 | (-3.0, 10.3) | 0.3 | (-4.4, 5.1) | 14.4 | (-4.9, 33.7) | -6.3 | (-24.3, 11.8) |
|  |  |  |  | |  | |  | |  | |  | |  | |

**Source**: Authors’ analysis of data from the National Health Interview Survey from 2016 to 2019. **Notes**: Study sample limited to adults ages 19–64 with income below 125 percent of the federal poverty level. Models adjusted for age, sex, race/ethnicity, education, marital status, citizenship, number of children, state-year unemployment rate, number of dentists per capita in each state, state-level COVID 19 rates during 2020, year, and state. All analyses used robust standard errors clustered by state. States that did and did not expand Medicaid during our study period and that do and do not provide adult Medicaid dental benefits are listed in eTable 1 in the Supplement. CI=confidence interval. ***p*< 0.05.

**eTable 5.** Sensitivity analyses according to state Medicaid expansion in the full sample and according to state’s dental benefits status.

|  | **Differences-in-Differences** | | | | | |
| --- | --- | --- | --- | --- | --- | --- |
|  | **Net change after 2020 (%, 95% CI)** | | | | | |
| **Outcome** | **Excluding states that expanded ACA in 2019,2020 ^a^** | | **Excluding states that changed their dental benefits ^b^** | | **Using alternative FPL cutoff ^c^** | |
| **Medicaid coverage** |  |  |  |  |  |  |
| Full sample | 2.4 | (-6.5, 11.2) | 2.9 | (-5.6, 11.4) | 3.4 | (-4.6, 11.4) |
| States without dental benefits | 7.7 | (-8.7, 24.2) | 6.2 | (-6.9, 19.2) | 5.7 | (-6.8, 18.2) |
| States with dental benefits | 0.5 | (-9.4, 10.4) | -0.1 | (-9.4,9.3) | 2.0 | (-7.3, 11.3) |
|  |  |  |  |  |  |  |
| **Uninsured** |  |  |  |  |  |  |
| Full sample | -5.6 | (-14.6, 3.4) | -6.1 | (-15.0, 2.9) | -8.1 | (-14.7, -1.4)** |
| States without dental benefits | -2.0 | (-23.0, 19.1) | -7.0 | (-24.3, 10.3) | -5.4 | (-17.8, 7.1) |
| States with dental benefits | -5.4 | (-17.13, 6.3) | -3.4 | (-14.4, 7.6) | -8.4 | (-17.8, 1.0) |
|  |  |  |  |  |  |  |
| **Medicaid dental coverage** |  |  |  |  |  |  |
| Full sample | 2.0 | (-2.0, 6.1) | 1.3 | (-1.8, 4.4) | 1.9 | (-2.2, 5.9) |
| States without dental benefits |  | -- |  | -- |  | -- |
| States with dental benefits | 0.5 | (-9.4, 10.4) | -0.1 | (-9.4, 9.3) | 2.0 | (-7.3, 11.3) |
|  |  |  |  |  |  |  |
| **Private dental insurance** |  |  |  |  |  |  |
| Full sample | 3.3 | (-1.3, 7.8) | 3.5 | (-0.8, 7.7) | 2.7 | (-1.7, 7.2) |
| States without dental benefits | -0.9 | (-10.3 , 8.4) | 0.8 | (-8.7, 10.3) | -0.1 | (-8.5, 8.2) |
| States with dental benefits | 1.7 | (-5.6, 8.9) | 3.1 | (-4.2, 10.4) | 1.8 | (-4.6, 8.2) |
|  |  |  |  |  |  |  |
| **No dental coverage** |  |  |  |  |  |  |
| Full sample | -5.7 | (-10.9, -0.5)** | -5.0 | (-9.8, -0.2)** | -5.0 | (-9.8, -0.3)** |
| States without dental benefits | 0.9 | (-8.4, 10.3) | -0.8 | (-10.3, 8.7) | 0.1 | (-8.2, 8.5) |
| States with dental benefits | -3.5 | (-14.6, 7.5) | -3.9 | (-14.9, 7.0) | -4.8 | (-13.9, 4.3) |
|  |  |  |  |  |  |  |
| **Seen a dentist in the past year** |  |  |  |  |  |  |
| Full sample | -13.1 | (-25.6, -0.6)** | -13.7 | (-25.0, -2.4)** | -12.2 | (-21.8, -2.7)** |
| States without dental benefits | -19.0 | (-47.8, 9.8) | -15.8 | (-42.3, 10.7) | -13.6 | (-28.2, 0.9) |
| States with dental benefits | -5.8 | (-24.8, 13.1) | -3.9 | (-23.1, 15.2) | -2.5 | (-20.8, 15.8) |
|  |  |  |  |  |  |  |
| **Couldn’t afford dental care in the past year** |  |  |  |  |  |  |
| Full sample | 5.7 | (-11.0, 22.4) | 4.8 | (-10.3, 19.8) | 3.5 | (-9.3, 16.4) |
| States without dental benefits | 4.6 | (-9.3, 18.5) | -1.4 | (-25.4, 22.6) | -5.8 | (-20.4, 8.8) |
| States with dental benefits | 11.3 | (-8.0, 30.7) | 9.1 | (-10.1, 28.4) | 8.0 | (-8.6, 24.6) |
|  |  | |  | |  | |

**Source**: Authors’ analysis of data from the National Health Interview Survey from 2016 to 2020. **Notes**: Study sample limited to adults ages 19–64 with income below 125 percent of the federal poverty level. ^a^Excluding states that expanded ACA in 2019 (ME, VA) and in 2020 (ID, UT, NE). ^b^Excluding states that changed their dental benefits during the study period between 2016 and 2020 (HI, ID, LA, WV). ^c^ Study sample limited to adults aged 19-64 years old with family income up to 138% of FPL using imputed family income files provided by the National Center for Health Statistics. Models adjusted for age, sex, race/ethnicity, education, marital status, citizenship, number of children, state-year unemployment rate, number of dentists per capita in each state, COVID-19 rates during 2020, year, and state. All analyses used robust standard errors clustered by state. States that did and did not expand Medicaid during our study period and that do and do not provide adult Medicaid dental benefits are listed in eTable 1 in the Supplement. CI=confidence interval. ***p*< 0.05.

**eTable 6.** Sensitivity analyses according to race and ethnicity.

|  | **Differences-in-Differences** | | | | | |
| --- | --- | --- | --- | --- | --- | --- |
|  | **Net change after 2020 (%, 95% CI)** | | | | | |
| **Outcome** | **Excluding states that expanded ACA in 2019,2020 ^a^** | | **Excluding states that changed their dental benefits ^b^** | | **Using alternative FPL cutoff ^c^** | |
| **Medicaid Coverage** |  |  |  |  |  |  |
| White | 0.5 | (-9.1, 10.2) | 2.8 | (-5.9, 11.5) | 4.4 | (-5.1, 13.8) |
| Black | -7.9 | (-29.0,13.2) | -6.4 | (-30.1, 17.4) | -4.6 | (-21.9, 12.7) |
| Hispanic | 3.8 | (-6.7,14.3) | 2.7 | (-7.4, 12.8) | 5.4 | (-2.5, 13.2) |
|  |  |  |  |  |  |  |
| **Uninsured** |  |  |  |  |  |  |
| White | -6.3 | (-13.8, 1.2) | -6.9 | (-14.4, 0.5) | -9.4 | (-17.0, -1.9)** |
| Black | 1.9 | (-17.0, 20.8) | 3.0 | (-16.8, 22.9) | -1.2 | (-18.1, 15.7) |
| Hispanic | -4.5 | (-15.0, 6.1) | -5.1 | (-15.4, 5.1) | -5.8 | (-13.2, 1.6) |
|  |  |  |  |  |  |  |
| **Medicaid dental Coverage** |  |  |  |  |  |  |
| White | -0.4 | (-6.7, 5.8) | -0.4 | (-5.2, 4.4) | 0.9 | (-5.7, 7.5) |
| Black | 0.7 | (-8.4, 9.8) | -0.9 | (-10.4, 8.6) | 0.7 | (-8.9, 10.2) |
| Hispanic | 0.1 | (-9.9, 10.2) | -0.1 | (-9.6, 9.3) | 2.4 | (-4.5, 9.4) |
|  |  |  |  |  |  |  |
| **Private dental insurance** |  |  |  |  |  |  |
| White | 7.6 | (0.5, 14.7)** | 7.1 | (-0.1, 14.3) | 3.5 | (-4.2, 11.3) |
| Black | -2.0 | (-14.5, 10.6) | -1.4 | (-15.0, 12.2) | -1.5 | (-12.9, 9.9) |
| Hispanic | 4.0 | (-5.6, 13.6) | 3.2 | (-5.7, 12.1) | 3.3 | (-4.6, 11.2) |
|  |  |  |  |  |  |  |
| **No dental coverage** |  |  |  |  |  |  |
| White | -7.9 | (-15.8, 0.046) | -7.4 | (-15.1, 0.2) | -5.1 | (-12.5, 2.3) |
| Black | 0.8 | (-10.7, 12.3) | 3.1 | (-8.5, 14.7) | 0.6 | (-10.1, 11.3) |
| Hispanic | -5.7 | (-17.3, 5.9) | -4.6 | (-15.7,6.5) | -6.5 | (-16.2, 3.1) |
|  |  |  |  |  |  |  |
| **Seen a dentist in the past year** |  |  |  |  |  |  |
| White | -11.1 | (-28.9, 6.7) | -13.3 | (-29.6, 2.9) | -12.0 | (-28.8, 4.8) |
| Black | -54.1 | (-97.8, -10.3)** | -41.7 | (-95.2, 11.7) | -32.6 | (-72.8, 7.6) |
| Hispanic | 23.7 | (0.8, 46.6)** | 16.6 | (-10.4, 43.7) | 10.4 | (-11.3 ,32.0) |
|  |  |  |  |  |  |  |
| **Couldn’t afford dental care in the past year** |  |  |  |  |  |  |
| White | 14.2 | (-7.1, 35.5) | 12.3 | (-6.3, 30.8) | 12.4 | (-5.4, 30.1) |
| Black | 27.9 | (-0.3, 56.0) | 16.4 | (-23.4, 56.1) | -4.0 | (-32.4, 24.4) |
| Hispanic | -23.5 | (-46.6, -0.4)** | -16.4 | (-38.7, 5.9) | -11.8 | (-32.6, 9.1) |
|  |  | |  | |  | |

**Source**: Authors’ analysis of data from the National Health Interview Survey from 2016 to 2020. **Notes**: Study sample limited to adults ages 19–64 with income below 125 percent of the federal poverty level. ^a^ Excluding states that expanded ACA in 2019 (ME, VA) and in 2020 (ID, UT, NE). ^b^ Excluding states that changed their dental benefits during the study period between 2016 and 2020 (HI, ID, LA, WV). ^c^ Study sample limited to adults aged 19-64 years old with family income up to 138% of FPL using imputed family income files provided by the National Center for Health Statistics. Models adjusted for age, sex, race/ethnicity, education, marital status, citizenship, number of children, state-year unemployment rate, number of dentists per capita in each state, COVID-19 rates during 2020, year, and state. All analyses used robust standard errors clustered by state. States that did and did not expand Medicaid during our study period are listed in eTable 1 in the Supplement. CI=confidence interval. ***p*< 0.05 ****p*< 0.01.

**Appendix 7. Methods**

**Regression equation - Differences in Differences Model (Table 1, eTable 3- 6)**

For each outcome *Y* we estimated the following linear probability model:

*Y_ist_=β_0_ + β_1_ Year2020_t_ + β_2_ Expansion State_s_ +β_3_ (Year 2020* ExpansionState)_st_ + X_ist_ + ε_ist_*

*Equation (1)*

Where *i* indexed individual, *s* state, and *t* year. *Year2020_t_* is an indicator variable for time t being after the COVID-19 pandemic, *State_s_* is an indicator variable for exposure group *s* , *X_ist_* represents individual level covariates and *ε_ist_* is the error term. *Year2020_t_* is equal to one if the observation occurs after COVID-19 pandemic. Expansion*State_s_* is equal to one if the observation is in a state that expanded Medicaid and equal to zero in a state that doesn’t expand Medicaid. β_1_ and β_2_ are vectors of time and state fixed effects respectively. Year fixed effects control for any secular trends in the outcome that are common across states. State fixed effects control for any unmeasured differences between states. The coefficient of interest is *β_3_* that represents the change in outcome in 2020 among those in Medicaid expansion states, compared to those living in states without the expansion. The model controls for Medicaid eligibility variables and other individual covariates as Xist including age, sex, race/ethnicity, education, marital status, citizenship, number of children, state-year unemployment rate, number of dentists per capita in each state, state-level COVID-19 rates during 2020, year, and state. All analyses used robust standard errors clustered by state.
